# Effect of Osteoking on the osteogenic and adipogenic differentiation potential of rat bone marrow mesenchymal stem cells in vitro

**DOI:** 10.1186/s12906-019-2435-6

**Published:** 2019-01-31

**Authors:** Congtao Yu, Lifen Dai, Zhaoxia Ma, Hongbin Zhao, Yong Yuan, Yunfeng Zhang, Pengfei Bao, Yanfang Su, Daiping Ma, Change Liu, Xingfei Wu, Jinxue Liu, Yanjiao Li, Bing Wang, Min Hu

**Affiliations:** 1 Yunnan Key laboratory for Basic Research on Bone and Joint Diseases, Kunming University, Kunming, 650214 China; 2 Second Affiliated Hospital of Kunming Medical University, Kunming, 650500 China; 3 Department of Emergency Surgery, The First People’s Hospital of Yunnan Province, Affiliated Hospital of Kunming University of Science and Technology, Kunming, 650032 China; 4 Bone and Joint Rehabilitation Department, Disabled Rehabilitation Centre of Yunnan Province, Kunming, 650032 China; 5 First Affiliated Hospital of Kunming Medical University, Kunming, 650031 China

**Keywords:** Osteoking, Mesenchymal stem cells, Osteogenic differentiation, Adipogenic differentiation, Bone disease

## Abstract

**Background:**

Bone damage is a condition that affects the quality of life of patients. Mesenchymal stem cells (MSCs) are important for bone repair. Osteoking is a natural compound in traditional Chinese Medicine used to treat bone diseases; however, the effect of Osteoking on the differentiation of MSCs has not been reported. In this study, we aimed to investigate the effect of Osteoking on the osteogenic and adipogenic differentiation potential of rat bone marrow mesenchymal stem cells (rbMSCs).

**Methods:**

The effects of Osteoking on the proliferation and differentiation of rbMSCs were investigated. Different concentrations of Osteoking were prepared, and its cytotoxicity was evaluated by CCK-8 assay. The expression of osteogenic and adipogenic genes were determined, and several staining methods were used to reveal the osteogenic and adipogenic differentiation potential of rbMSCs.

**Results:**

Our results show that appropriate concentrations of Osteoking can enhance osteogenic differentiation of rbMSCs and reduce adipogenic differentiation without any effect on proliferation. This may be related to the changes in related gene expression.

**Conclusion:**

Osteoking enhances osteogenic differentiation and inhibits adipogenic differentiation of rbMSCs. Therefore, Osteoking may have a therapeutic potential for treating bone disease caused by changes in differentiation function of MSCs.

## Background

Many people suffer from different kinds of bone diseases [1, 2]. Although bone tissue is a vascularized tissue with the ability to heal itself, the repair outcome and time taken for repair did not reach satisfying levels [3, 4]. Osteoblasts derived from mesenchymal stem cells (MSCs) play a central role in bone formation and remodeling [5, 6]. However, MSCs do not always differentiate into osteoblasts because of their multiple differentiation potential, especially in the elderly, whose MSCs have weak osteogenic capacity but good adipogenic capacity [7–9]. Variation in differentiation capacity may lead to bone diseases such as osteoporosis [10]. Therefore, regulating the differentiation ability of MSCs is necessary.

Osteoking, also known as Heng-Gu-Gu-Shang-Yu-He-Ji, is a natural compound in traditional Chinese Medicine (TCM) extracted from Chinese herbs, including *Pericarpium Citri reticulatae, Carthamus tinctorius L, Radix notoginseng, Eucommia ulmoides Oliv, Radix ginseng, Radix Astragali Mongolici and Carapax trionycis*, which have been used to treat bone diseases for thousands of years in Yunnan Province, China. Osteoking was approved by the Chinese State Food and Drug Administration in 2002 and exhibits curative effects in the clinical treatment of different kinds of bone diseases, especially femoral head necrosis, prolapse of the lumbar intervertebral disc, fracture, and osteoarthritis [11]. Although single molecules have high efficiency and few side effects, they could not achieve satisfactory effects because bone repair is a complex process. Osteoking has multi-effects on bone repair. Previous studies by our group demonstrated that Osteoking has the ability to regulate the expression of several genes related to bone formation and angiogenesis [12, 13]. Clinical studies have demonstrated that Osteoking has achieved good curative effects in preventing fracture and treating ischemic necrosis of the femoral head in humans [14]. However, the mechanism of bone repair by Osteoking at cellular level has not been reported previously. MSCs, which could differentiate into osteoblasts, are one of the most important cells related to bone repair. Previous studies have shown that aging or unhealthy MSCs have weakened osteogenic differentiation capacity and enhanced adipogenic differentiation capacity, which is a possible cause of osteoporosis in the elderly [15–18]. Considering the beneficial effects of Osteoking in the treatment of bone disease, it is possible that Osteoking can enhance the osteogenic differentiation potential and inhibit the adipogenic differentiation potential of MSCs. To test this hypothesis, we investigated the effect of different concentrations of Osteoking on the osteogenic and adipogenic differentiation potential of rat bone marrow MSCs (rbMSCs) in vitro in this study.

## Methods

### Animals

Three-month-old male Sprague Dawley (SD) rats were purchased from Kunming University Laboratory Animal Center. The animal handlings and experimental procedures were approved by the Medical Ethics Committee of Medicine Department of Kunming University.

The rats were sacrificed by 3% pentobarbital sodium in a dose of 150 mg/kg and disinfected with 75% ethanol. The femur and tibia were removed and washed with phosphate-buffered saline (PBS) three times in a sterile environment. The osteoepiphyses were removed to acquire the bone marrow.

### Different concentration Osteoking preparation

The Osteoking concoction was prepared according to the Chinese Pharmacopeia (China Pharmacopeia Committee, 2002) and was supplied by Yunnan Crystal Natural Pharmaceutical Co.,Ltd. (Kunming, China) [19]. Briefly, *Pericarpium Citri Reticulatae* (10 g), *Carthamus tinctorius* L. (15 g), *Radix Notoginseng* (30 g), *Eucommia ulmoides* Oliv. (30 g), *Radix Ginseng* (20 g), *Radix Astragali Mongolici* (40 g), and *Carapax Trionycis* (10 g) were broken into coarse powder and immersed in 10× (*V*/*W*) distilled water for 12 h at room temperature, and then boiled in a distillation apparatus for 1 h. This process was repeated twice, and for the second and third extraction, the residue from the previous extraction was filtered, and the same extracting condition was applied. Thereafter, the combined extracts were filtrated and evaporated using a rotary evaporator at 50 °C to a relative density of 1.03–1.04, centrifuged for 30 min at 12,000 rpm and the supernatant obtained was centrifuged once again after standing for 12 h. In the end, adjusted the pH value to 4.0–6.0, added distilled water to a total volume of 1000 mL and filtrated for usage. The crude drug concentration is 0.36 g/ml. 50X represent 1 ml Osteoking were diluted with H-DMEM to 50 ml. 50X was diluted 5 times by H-DMEM to prepare 250X, and so on. The H-DMEM with Osteoking was used for cell differentiation and cytotoxicity assay.

Identification of all plant materials used in this study were undertaken by Yunnan Crystal Natural Pharmaceutical Co.,Ltd. according to the Chinese Pharmacopeia (2015 Edition). The inspection report numbers are Y-02-201,512,023 (*Pericarpium Citri Reticulatae*), Y-02-201,512,021 (*Carthamus tinctorius L*), Y-02-201,512,020 (*Radix Notoginseng*), Y-02-201,512,022 (*Eucommia ulmoides Oliv*), Y-02-201,512,019 (*Radix Ginseng*), Y-02-201,512,025 (*Radix Astragali Mongolici*), Y-02-201,512,026 (*Carapax Trionycis*) respectively.

### Cell culture and differentiation

rbMSCs were obtained from the bone marrow of adult male rats. The cells were seeded in basal medium containing L-DMEM (Hyclone, USA) and 8% fetal bovine serum (FBS, BI, USA) and cultured at 37 °C with 5% CO_2_. Medium was changed every other days and cells were passaged when the cell confluence was about 90%. Cells were digested with 0.25% pancreatin at 37 °C for 2 min.

P3 rbMSCs were seeded onto 24-wells (Corning, USA) beginning with 5 × 10^4^ cells per well for differentiation. After culturing in basal medium for 12 h, differentiation was initiated by using specific media. The osteoblast media (OB) contained H-DMEM (Hyclone, USA), 10% FBS (BI, USA), 100 nM/L dexamethasone (Sigma, USA), 10 mM/L β-glycerophosphate (Sigma, USA), and 0.2 mM/L ascorbate-2-phosphate (Sigma, USA), while the adipocyte media (AD) contained H-DMEM (Hyclone, USA), 10% FBS (BI, USA), 1 μM/L dexamethasone (Sigma, USA), 0.5 mM/L isobutylmethylxanthine (Sigma, USA), 200 μM/L indomethacin (Sigma, USA), and 10 mg/L insulin (Sigma, USA). Treatment with Osteoking was initiated at the same time as the differentiation process. Different concentrations of Osteoking (Yunnan Crystal Natural Pharmaceutical Co., Ltd., China) were added to the respective media. 50X, 250X, 1250X, 6250X, and 31,250X represent rbMSCs cultured in media with original OsteoKing liquid diluted 50X, 250X, 1250X, 6250X, and 31,250X times, respectively. The Control which was used as negative control represents rbMSCs treated with only media, Control-OB and Control-AD which were used as positive control represents rbMSCs treated with the osteoblast media and the adipocyte media. For qRT-PCR, the cells were harvest when the cell confluence was about 90%.

### Immunofluoresent microscopy and flow cytometric analysis

Cells plated on 24-well were fixed by 4% PFA solution for 10 min and then changed to PBS at room temperature. Cells were then treated with 0.1% Triton X-100 for 10 min, followed by incubation in blocking buffer (3% bovine serum albumin in PBS) for 30 min. Afterwards, samples were incubated with primary antibodies at 4 °C overnight and then with appropriate fluorescent probe-conjugated with secondary antibodies for 2 h at RT. Nuclei were counter-stained with DAPI. Images were captured with fluorescence microscope (Nikon).

### In vitro cytotoxicity assays

Cell viability was assessed with the Cell Counting Kit-8 (Beyotime Biotechnology, China). Absorbance was measured on an enzyme-linked immunosorbent assay (ELISA) plate reader (Infinite M200 Pro, Tecan, Germany).

### Histochemical staining

To confirm osteogenesis, cells cultures in osteogenic media (OM) were stained using 1-step AP staining kits (SiDanSai, China) or Alizarin red (sigma, USA). The cells were fixed in 4% paraformaldehyde for 5–10 min, washed with PBS, mixed the 1-step AP or Alizarin red until desired stain developed. Then the cells were rinsed with PBS and viewed under a light microscope.

To confirm adipogenesis, the cells cultured in adipogenic media (AM) were stained using Oil Red O (sigma, USA). The cells were fixed in 4% paraformaldehyde for 5–10 min, washed with PBS, and incubated with Oil Red O solution for 20 min at 20 °C. Then, the cells were rinsed with PBS and viewed under a light microscope.

### Quantitative real-time PCR

Total RNA samples were collected with Trizol (TaKaRa) according to manufacturer’s instructions. 1.0 μg RNA for each reaction was reverse-transcribed to cDNA. Quantitative real-time PCR was subsequently conducted with specific primers and SYBR Green (TaKaRa) in Applied Biosystems 7900HT Fast Real-Time PCR System. Forward used at a final concentration of 400 nM and reverse used at a final concentration of 400 nM. Cycling for gene PCR: denature at 95 °C for 15 s, anneal at 60 °C for 15 s, extend at 72 °C for 20s, hold at 72 °C for 7 min with data collection, 40 cycles. The relative quantities of the gene tested per sample were calculated against 36B4 using the △△C(T) formula as previously described [20]. Primers used were listed in Table 1.

**Table 1:** Primers for qRT-PCR

Target gene	Primer(5′-3′)	GenBank Accession
PPARγ	F:5′- ATGACAGACCTCAGGCAGATTG − 3′	XM_006237009
	R:5′- GTCAGCGACTGGGACTTTTCT − 3′	
PLIN1	F:5′- ATGAGGAGGAAGAAGAAGAAGAGTC -3′	XM_008759499
	R:5′- TCAGGGCATCGGATAGGGA − 3′	
OSX	F:5′- GTTCACCTGTCTGCTCTGCT − 3’	XM_006242390
	R:5′- TTGGCTTCTTCTTCCCCGAC -3’	
ALP	F:5′- CCCACAAGAGCCCACAATGG − 3’	XM_006239136
	R:5′- CGGAAGTGAGGCAGGTAGCAAA − 3’	
actin	F:5′- ACCGTGAAAAGATGACCCAGAT − 3’	NM_031144
	R:5′- CCAGAGGCATACAGGGACAACA − 3’	
OCN	F:5′- GAGGGCAGTAAGGTGGTGAATAG − 3’	NM_013414.1
	R:5′- AAGCCAATGTGGTCCGCTAG-3’	
BSPII	F:5′- CAACGGCACCAGCACCAA-3’	NM_012587.2
	R:5′- TCGTATTCTTCCCCATACTCAACC-3’	
COL2A1	F:5’-TAAAACCCTCAACCCCAAAACA-3’	NM_012929.1
	R:5′- ATCAGGTCAGGTCAGCCATTCA-3’	

### Statistical analysis

Student’s *t*-test was used for simple two-sample comparisons. One-way analysis of variance (ANOVA) with post-hoc Tukey HSD test was used for multiple comparisons between different sample groups. A *p*-value < 0.05 was considered statistically significant.

## Results

### Establishment of rbMSCs

**Fig. 1:**
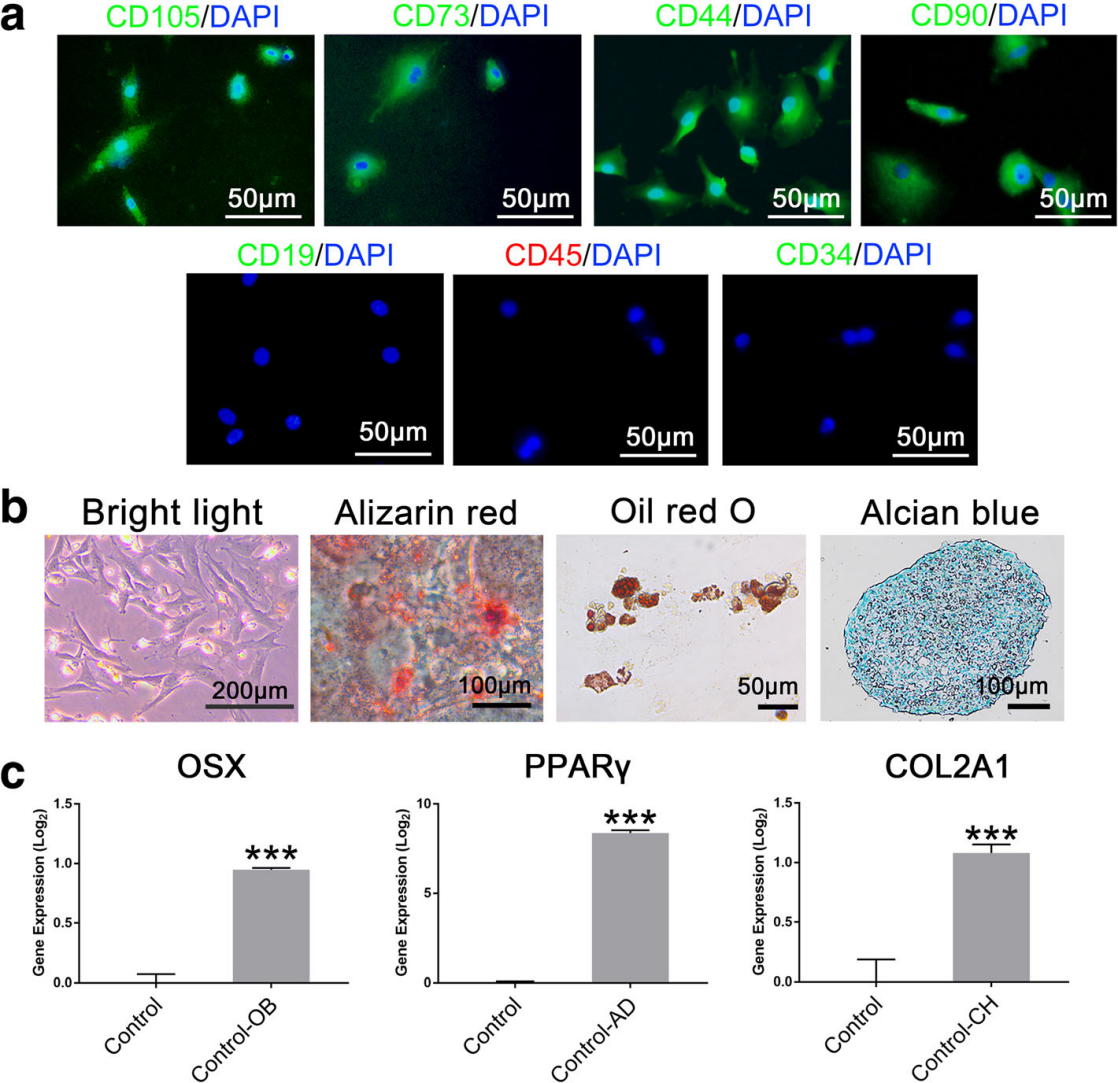
Identification of rat bone marrow mesenchymal stem cells. **a** Dual-staining of rat bone marrow mesenchymal stem cells (rbMSCs) with CD44/DAPI, CD19/DAPI, CD45/DAPI, CD105/DAPI, CD73/DAPI, CD34/DAPI and CD90/DAPI. **b** Cell morphology before induction in bright light. Three-lineage differentiation of rat mesenchymal stem cells. Osteogenic differentiation is determined by Alizarin red staining, adipogenic differentiation is determined by Oil Red O staining, and chondrogenic differentiation is identified by Alcian blue staining. **c** qRT-PCR analysis of OSX levels upon osteogenic differentiation,, PPARγ upon adipose differentiation and COL2A1 levels upon chondrogenic differentiation. ***p < 0.001, *n* = 3, when samples were compared to Control group

We established the rat bone mesenchymal stem cell line from rat bone tissue. To identify the cultured cells further, they were stained with CD44/DAPI, CD19/DAPI, CD45/DAPI, CD105/DAPI, CD73/DAPI, CD34/DAPI and CD90/DAPI (Fig. 1a). The results indicated that these cells showed CD44, CD105, CD73 and CD90 positive expression while CD19, CD34 and CD45 negative expression according to the characteristics of MSCs (Fig. 1a). The rbMSCs were in a MSC-like form, with a long fusiform shape. In addition, the three-lineage differentiation capacity of rbMSCs was evaluated by staining and qRT-PCR (Fig. 1b). Gene expression of OSX, PPARγ and COL2A1 for three lineage was obviously higher than cells before corresponding induction (Fig. 1c). The results showed that rbMSCs could differentiate into osteoblasts, adipocytes and chondrocytes. Thus, we established the rat bone mesenchymal stem cell line successfully.

### High concentration of Osteoking induced cytotoxicity on rbMSCs

**Fig. 2:**
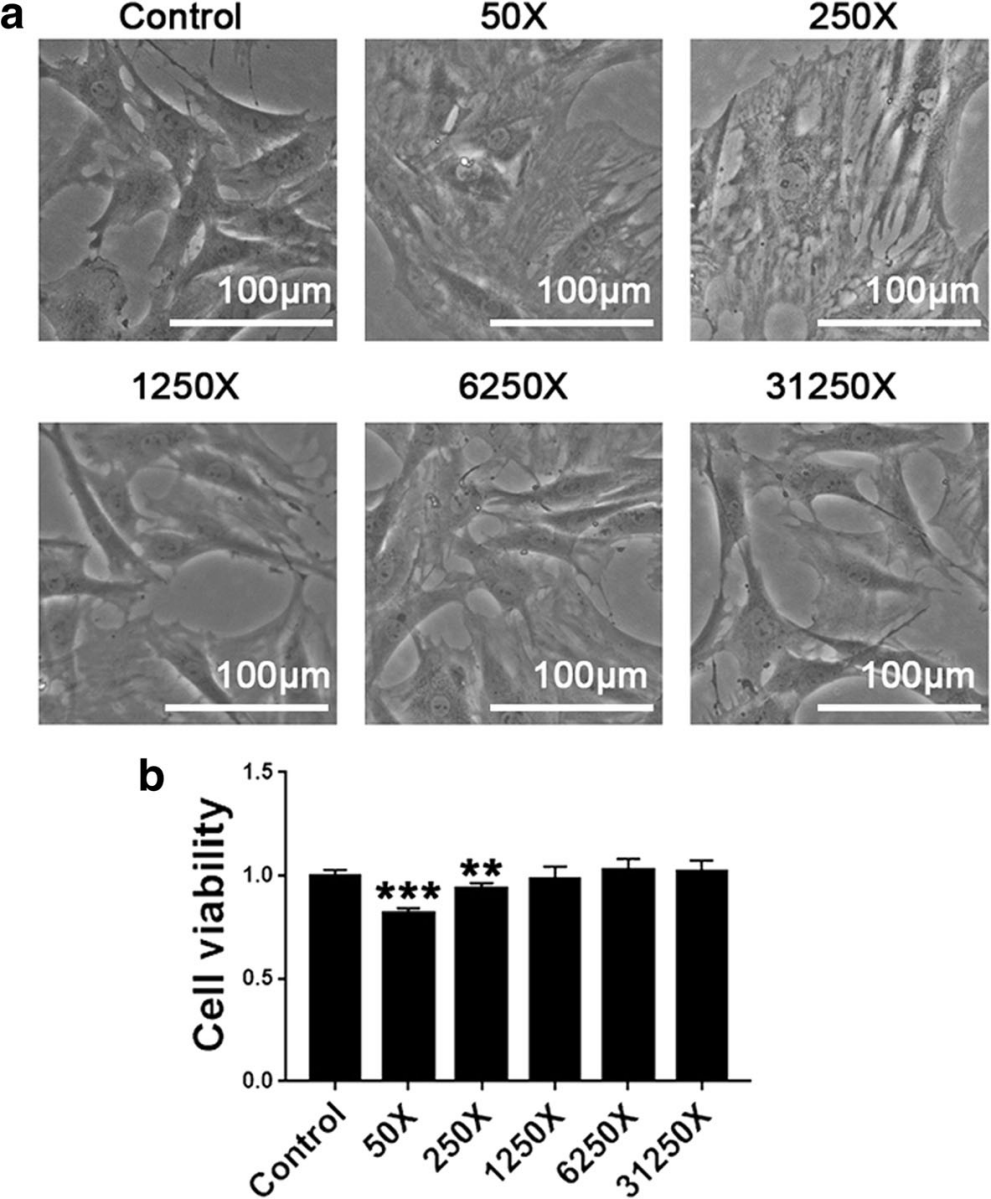
Evaluation of cytotoxicity of different concentrations of Osteoking on rat bone marrow mesenchymal stem cells. **a** Cell morphology after culturing with different concentrations of Osteoking for 4 days in bright light. X = dilution multiple of Osteoking. **b** Cell viability of rat bone marrow mesenchymal stem cells (rbMSCs) treated with different concentrations of Osteoking after 4 days by CCK-8 assay. **p < 0.01, ***p < 0.001 vs. control; *n* = 5

We prepared different concentrations of Osteoking to evaluate the cytotoxicity on rbMSCs. High concentrations of Osteoking had a negative impact on the growth and morphology of rbMSCs in bright light (Fig. 2a). To evaluate the cytotoxicity of Osteoking, CCK-8 assay was used for quantitative research. The result showed that 50X exhibited high cytotoxicity against rbMSCs, while 250X also exhibited cytotoxicity to a certain extent (Fig. 2b). Other concentrations did not exhibit obvious cytotoxicity when compared with the control group.

### Appropriate concentrations of Osteoking promoted osteogenic differentiation of rbMSCs

**Fig. 3:**
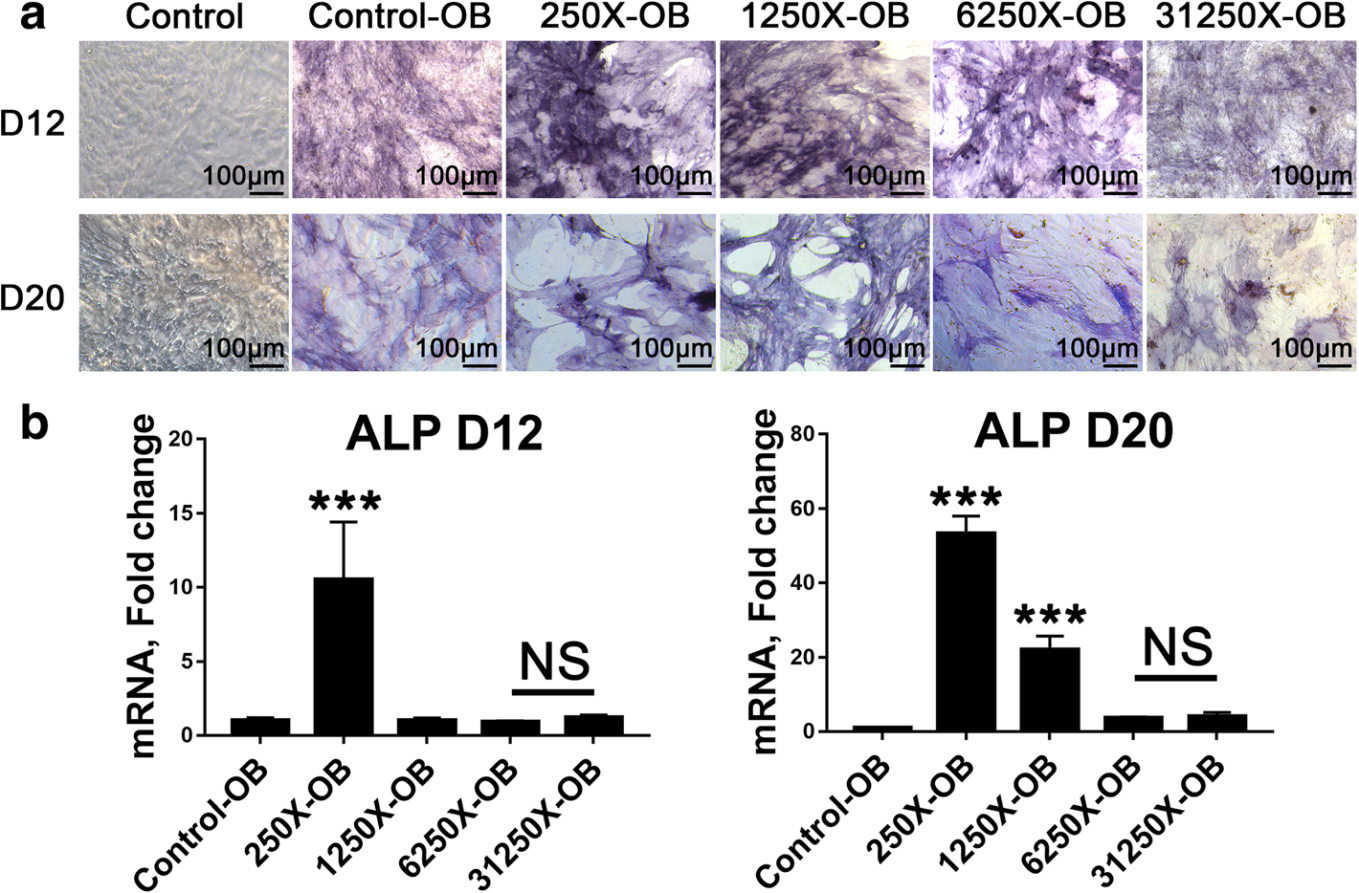
Effect of different concentrations of Osteoking on alkaline phosphatase expression in rbMSCs. **a** Alkaline phosphatase (ALP) staining of rat bone marrow mesenchymal stem cells (rbMSCs) cultured in mesenchymal stem cell (MSC) medium or osteoblast maturation medium with different concentrations of Osteoking after 12 and 20 days. D = day, X = dilution multiple of Osteoking. **b** Gene expression analysis for ALP of rbMSCs cultured in osteoblast maturation medium with different concentrations of Osteoking after 12 and 20 days by qRT-PCR., ***p < 0.001 vs. control; *n* = 5

The level of alkaline phosphatase (ALP), an early marker of osteogenesis, was determined to show the osteogenesis status by staining and qRT-PCR (Fig. 3). The control represents rbMSCs treated with only MSC culture media for 12 or 20 days; control-OB represents rbMSCs cultured in osteoblast media for 12 or 20 days; and 250X-OB, 1250X-OB, 6250X-OB, and 31,250X-OB were cultured in osteoblast media containing original OsteoKing liquid diluted the corresponding number of times for 12 or 20 days. The results showed that 250X-OB significantly increased the expression of ALP at mRNA and protein levels. However, after 20 days, ALP staining of 250X-OB showed that some rbMSCs died, which is in agreement with the results of cytotoxicity experiment. 1250X-OB also exhibited marked enhancement in ALP expression without toxicity. 6250X-OB and 31,250X-OB did not exhibit enhancement of ALP expression relative to Control-OB.

**Fig. 4:**
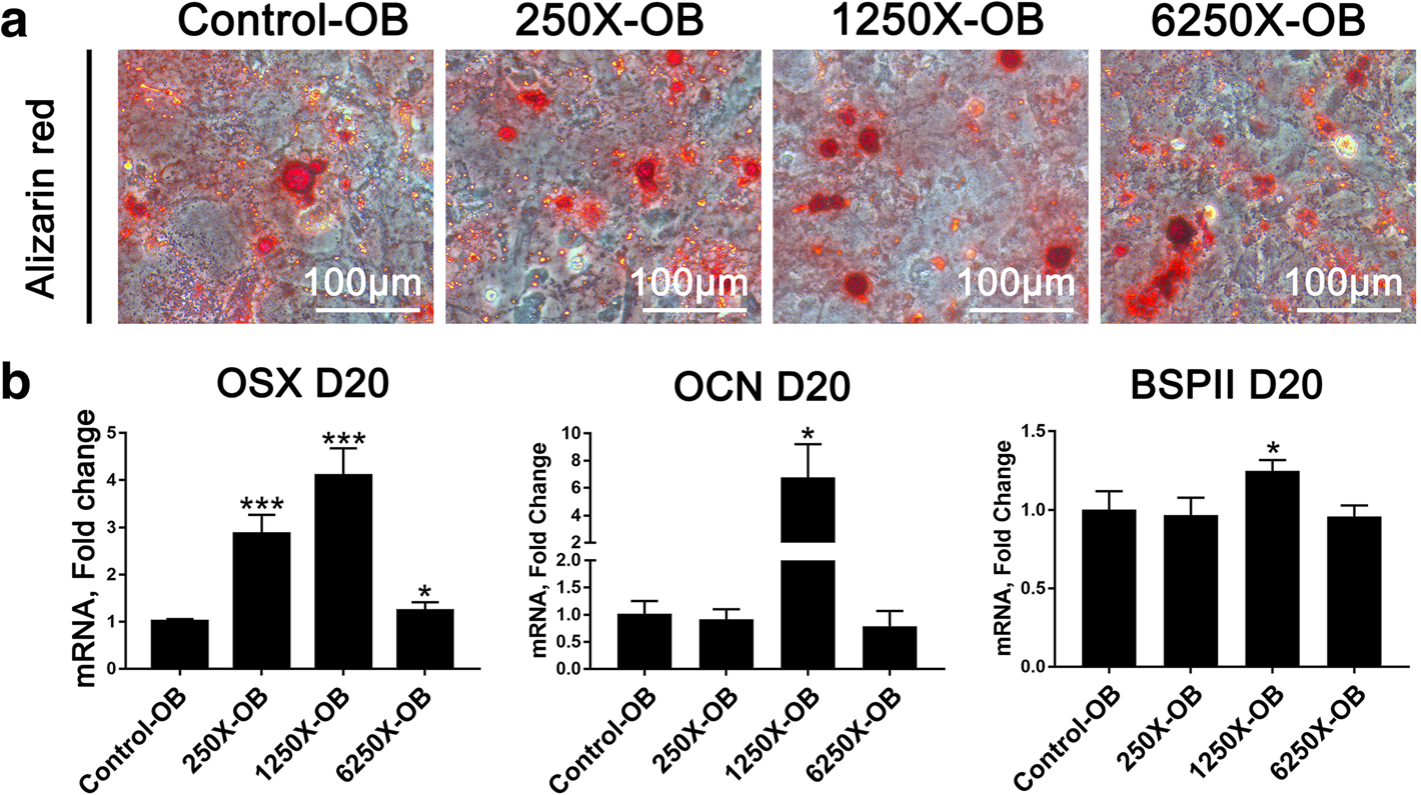
Effect of different concentrations of Osteoking on osteogenic differentiation of rbMSCs. **a** Alizarin red staining of rat bone marrow mesenchymal stem cells (rbMSCs) cultured in osteoblast maturation medium with different concentrations of Osteoking after 20 days. X = dilution multiple of Osteoking. **b** Gene expression analysis for osterix (OSX), bone Sialoprotein II (BSPII) and Osteocalcin (OCN) in rbMSCs cultured in osteoblast maturation medium with different concentrations of Osteoking after 20 days by qRT-PCR. *p < 0.05, ***p < 0.001 vs. control; *n* = 5

Based on the results of ALP analysis, we selected four groups for subsequent experiments. Alizarin red staining was used to show calcium deposition, and the gene expression of osterix (OSX), bone Sialoprotein II (BSPII) and Osteocalcin (OCN), some important osteogenesis genes, were determined. 1250X-OB exhibited the best osteogenic differentiation capacity among the four groups both in staining results and gene expression level (Fig. 4a and b). Although 250X-OB also increased the gene expression of OSX, it exhibited cytotoxicity on rbMSCs, so Alizarin red staining did not show better osteogenic differentiation capacity than Control-OB (Fig. 4a and b).

### Osteoking reduced the adipogenic differentiation potential of rbMSCs

**Fig. 5:**
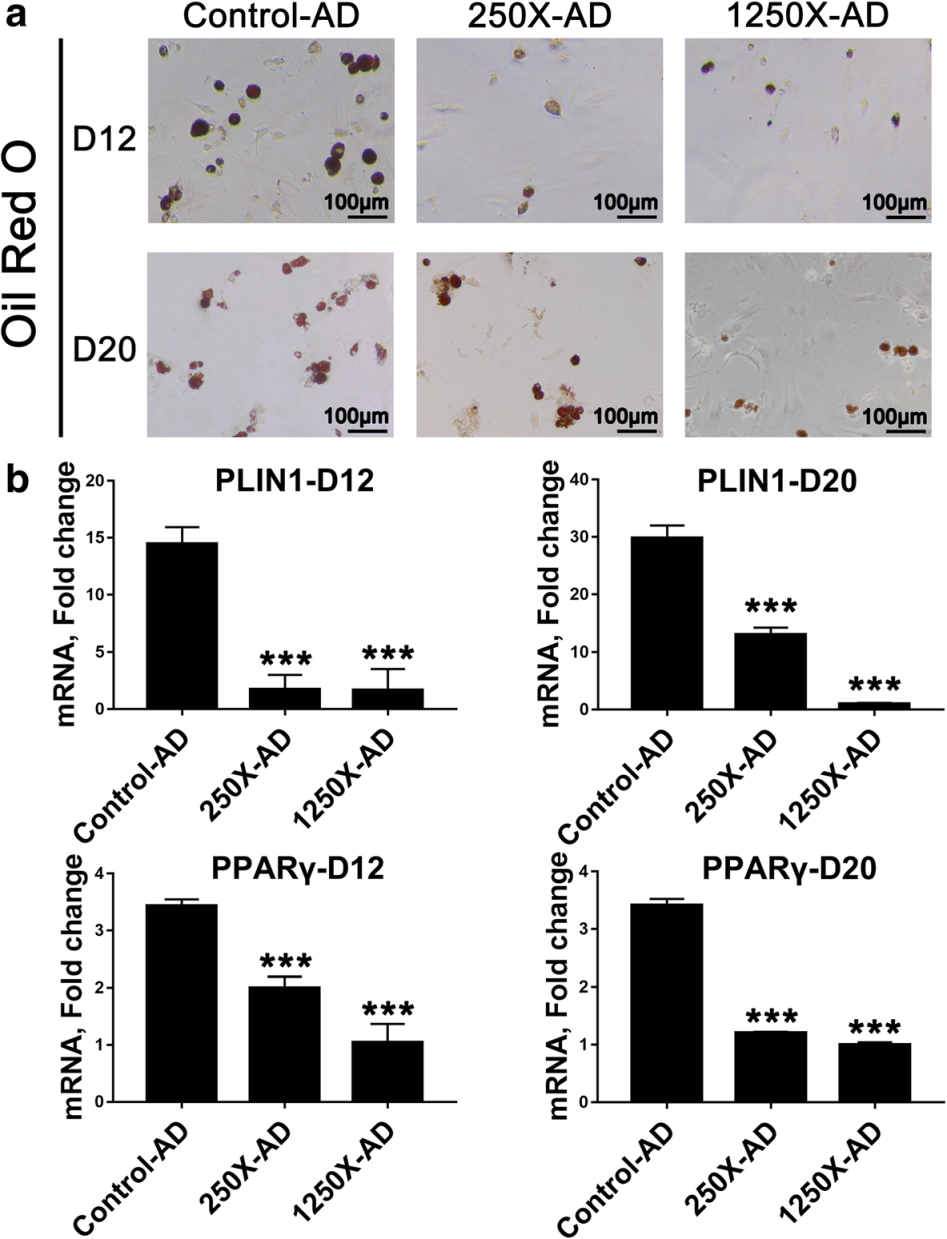
Effect of different concentrations of Osteoking on adipogenic differentiation potential of rbMSCs **a** Oil Red O staining of rat bone marrow mesenchymal stem cells (rbMSCs) cultured in adipose medium with different concentrations of Osteoking after 12 days and 20 days. D = day, X = dilution multiple of Osteoking. **b** Gene expression analysis for key adipogenic markers by qRT-PCR. ***p < 0.001 vs. Control; *n* = 4

The process of osteogenic differentiation is opposite to that of adipogenic differentiation; therefore, we evaluated the effect of Osteoking 250X and 1250X, which have clear effects on osteogenesis differentiation of rbMSCs. Oil Red O was used to determine adipogenic differentiation. The expression of PPARγ and PLIN1, two important adipogenic genes, were analyzed by qRT-PCR (Fig. 5). Osteoking 250X and 1250X could remarkably inhibit the adipogenic differentiation of rbMSCs (Fig. 5a), and the gene expression of PPARγ and PLIN1 was downregulated (Fig. 5b).

## Discussion

Many orthopedic diseases are related to the characteristic variation of MSCs. For example, MSCs of osteoporosis rats obviously decreased in osteogenic differentiation compared to normal [21]. The use of Osteoking in the treatment of bone diseases has shown good results in clinical settings. Previous study have established some possible mechanisms. For example, osteoking could up-regulate the expression of RUNX2 and VEGF which may enhance the bone formation and vascular regeneration [12, 13]. But its effect on MSCs has not been elucidated. In this study, we aimed to investigate the effect of different concentrations of Osteoking on the osteogenic and adipogenic differentiation potential of rbMSCs in vitro. We found that Osteoking could enhance osteogenic differentiation and inhibit adipogenic differentiation of rbMSCs.

In this study, we showed that Osteoking can increase the expression of osteogenesis gene and promote osteogenic differentiation. It is important to note that very high concentrations of Osteoking exhibits significant cytotoxicity on rbMSCs. From ALP, the early marker of osteogenic differentiation, the effect of Osteoking in promoting osteogenesis differentiation seems to be dose-dependent. But from OCN and BSPII, late markers of osteogenic differentiation, the osteogenic differentiation ability in high concentration group (250X) is not stronger than that in appropriate concentration (1250X). We suggested that the toxicity caused by high concentration Osteoking may influence the cell viability and differentiation. Such results indicate that excessive dose may enhance the toxicity without better curative effect. We selected an appropriate concentration that does not show cytotoxicity but demonstrates strong enhancement of osteogenic differentiation. This concentration agrees well with the dosage used in clinical settings, considering absorption and metabolism. These results indicates that the dosage of Osteoking should be strictly controlled to ensure the safety and effective in clinical treatment. At this concentration, the adipogenic differentiation of rbMSCs was also significantly inhibited. Interestingly, previous study reported that OSX represses adipogenesis by negatively regulating PPARγ, our results are very consistent with this [22]. 1250X-OB showed the most significant enhancement in OSX while 1250X-AD showed the most significant inhibition in PPARγ, this indicates that OSX may be a key gene for Osteoking to regulate MSC osteogenic and adipogenic differentiation. More extensive mechanism is to be studied further.

The osteogenic differentiation capacity of MSCs may be affected by various reasons such as aging and chemotherapy. This would subsequently lead to osteoporosis, fracture, and other bone diseases. Osteoking, as TCM, has complex constituents and multiple function. Evaluating its specified function by cell model could provide multiple information including cytotoxicity and function which can support the safety and effectiveness. The results of this study provide evidence for the potential of Osteoking in the treatment of bone disease and indicate that Osteoking may be used in the treatment for other disease caused by the weakened differentiation potential of MSCs.

## Conclusions

Overall, our study demonstrated that Osteoking could enhance osteogenic differentiation and inhibit adipogenic differentiation of rbMSCs. This finding provides theoretical basis for extensive use of Osteoking in clinical settings and the potential of Osteoking as a new agent to treat bone diseases caused by the decreasing osteogenic differentiation capacity of MSCs.

## Abbreviations

MSC: Mesenchymal stem cell
rbMSC: Rat bone marrow mesenchymal stem cell
ALP: Alkaline phosphatase
OSX: Osterix
PPARγ: Peroxisome proliferator-activated receptor gamma
PLIN1: Perilipin-1
TCM: Traditional Chinese Medicine
